# Sevoflurane-Induced Neuroapoptosis in Rat Dentate Gyrus Is Activated by Autophagy Through NF-κB Signaling on the Late-Stage Progenitor Granule Cells

**DOI:** 10.3389/fncel.2020.590577

**Published:** 2020-12-15

**Authors:** Dongyi Tong, Zhongliang Ma, Peng Su, Shuai Wang, Ying Xu, Li Min Zhang, Ziyi Wu, Kun Liu, Ping Zhao

**Affiliations:** ^1^Department of Anesthesiology, Shengjing Hospital of China Medical University, Shenyang, China; ^2^Department of Otolaryngology Head and Neck Surgery, Shengjing Hospital of China Medical University, Shenyang, China; ^3^Medical Research Center, Shengjing Hospital of China Medical University, Benxi, China; ^4^Department of Rehabilitation Medicine, The First Affiliated Hospital of Sun Yat-sen University, Guangzhou, China; ^5^Department of Anesthesiology, Cangzhou Central Hospital, Cangzhou, China

**Keywords:** sevoflurane, dentate gyrus, apoptosis, autophagy, NF-κB, differentiation

## Abstract

**Objective:**

The mechanisms by which exposure of the late-stage progenitor cells to the anesthesia sevoflurane alters their differentiation are not known. We seek to query whether the effects of sevoflurane on late-stage progenitor cells might be regulated by apoptosis and/or autophagy.

**Methods:**

To address the short-term impact of sevoflurane exposure on granule cell differentiation, we used 5-bromo-2-deoxyuridine (BrdU) to identify the labeled late-stage progenitor granule cells. Male or female rats were exposed to 3% sevoflurane for 4 h when the labeled granule cells were 2 weeks old. Differentiation of the BrdU-labeled granule cells was quantified 4 and 7 days after exposure by double immunofluorescence. The expression of apoptosis and autophagy in hippocampal dentate gyrus (DG) was determined by western blot and immunofluorescence. Western blot for the expression of NF-κB was used to evaluate the mechanism. Morris water maze (MWM) test was performed to detect cognitive function in the rats on postnatal 28–33 days.

**Results:**

Exposure to sevoflurane decreased the differentiation of the BrdU-labeled late-stage progenitor granule cells, but increased the expression of caspase-3, autophagy, and phosphorylated-P65 in the hippocampus of juvenile rats and resulted in cognitive deficiency. These damaging effects of sevoflurane could be mitigated by inhibitors of autophagy, apoptosis, and NF-κB. The increased apoptosis could be alleviated by pretreatment with the autophagy inhibitor 3-MA, and the increased autophagy and apoptosis could be reduced by pretreatment with NF-κB inhibitor BAY 11-7085.

**Conclusion:**

These findings suggest that a single, prolonged sevoflurane exposure could impair the differentiation of late-stage progenitor granule cells in hippocampal DG and cause cognitive deficits possibly via apoptosis activated by autophagy through NF-κB signaling. Our results do not preclude the possibility that the affected differentiation and functional deficits may be caused by depletion of the progenitors pool.

## Introduction

Sevoflurane is widely used as the sole anesthetic agents in pediatric surgical procedures because it is less neurotoxic than other volatile anesthetics, such as isoflurane (Drobish et al., 2016; Liu et al., 2017; Murphy et al., 2017). However, there is ongoing concern about its both short- and long-term effects on brain structure and cognitive function in younger patients. One prospective clinical study showed that <1 h of general anesthetic (GA) with sevoflurane is not associated with cognitive impairment compared to awake spinal anesthesia (SA) at 2 or 5 years of age (Davidson et al., 2016; McCann et al., 2019); however, whether longer exposure of sevoflurane is safe remains uncertain. Many basic and clinical studies have demonstrated that anesthetic neurotoxicity is determined by the stage of brain development at the time of exposure, the degree of anesthetic exposure, and regions of the brain (Hofacer et al., 2013; Qiu et al., 2016; McCann and de Graaff, 2017). An FDA warning announced that children < 3 years old who are exposed to anesthetics for >3 h may experience adverse impacts on brain development. In humans, postnatal neurogenesis peaks within the first 3 years of life, and this corresponds to within 14 days after birth in rodents. Therefore, in the basic rodent studies, neurotoxicity of GAs is widely measured within 14-day-old animals (Zhu et al., 2010). However, Hofacer et al. (2013) suggest that certain brain regions, such as dentate gyrus (DG) of the hippocampus, may be affected by 1.5% isoflurane even beyond the first 14 days of rodent life. In 21-day-old mice, studies have indicated that a large percentage of newly-born hippocampal DG cells undergo apoptosis. Especially, 14-day-old granule cells are vulnerable to cell death after anesthetic exposure in 21-day-old mice (Hofacer et al., 2013).

It is known that adult neurogenesis and synaptogenesis occur in the DG, and the integration of the newborn dentate granule cells (DGCs) plays important roles in cognitive and behavioral functions (Stratmann et al., 2010). Kang et al. (2017) found early postnatal exposure to isoflurane on P18 in mice caused cognitive deficits as early as P30, which corresponds to school-aged human children. In a previous study, we demonstrated that in juvenile animals (P21), the damage of 14-day-old hippocampal DGCs, which were sensitive to isoflurane-induced neuroapoptosis, recovered 2 months later (Jiang et al., 2016; Tong et al., 2019). However, we did not explore the short-term toxic effects on neurogenesis, or the cognitive and behavioral functions after sevoflurane exposure in those studies. Progenitor cells are continuously formed in the subgranular zone (DGZ) of the DG. These newborn granule cells promote neuronal differentiation, differentiated into mature granule cells, and integrated into the DG (Schaeffer et al., 2009; Cahill et al., 2017; Danzer, 2019). The differentiation and survival of new born cells are intimately controlled by apoptosis. Indeed, the most common outcome of GA exposure is apoptotic cell death (Istaphanous et al., 2013). Previously, isoflurane treatment on P21 rodents did not appear to affect the differentiation rate in hippocampal neurogenesis measure 2 months after exposure (Jiang et al., 2016). Based on these findings, we were interested in whether short-term (before 2 weeks) exposure to 3% sevoflurane would negatively affect adult hippocampal neurogenesis and cognitive changes. We especially wanted to determine if the process of differentiation from 14-day-old DGCs and the role of apoptosis have following exposure to sevoflurane.

The role of autophagy in the regulation of apoptosis and modulation of the neural stem cells proliferation and differentiation has been previously reported (Wang et al., 2018). However, whether autophagy following GA treatment may also modulate excessive apoptosis and differentiation of 14-day-old DGCs is not known. In the present study, we hypothesized that, at an anesthetic vulnerability period in rodents, excessive apoptosis induced by 3% sevoflurane is modulated by autophagy and this link between autophagy and apoptosis could give rise to changes in the differentiation, survival, and maturation of 14-day-old DGCs. The results from this study would provide vital insights into the role of apoptosis induced by anesthesia in adult neurogenesis during a crucial period when cognitive and learning abilities develop.

## Materials and Methods

### Animals

All procedures involving animals were approved by the Institutional Animal Care and Use Committee of the Shengjing Hospital Research Foundation (Approval No. 2016PS138K). All animals were maintained on a Sprague–Dawley background (Changsheng Co., Ltd., Shenyang, China, SCXK2015-0001). Sprague–Dawley rats were raised in a temperature- and humidity-controlled room with a 12:12 h light:dark cycle. The rats had free access to water and food. P21 rats (40–50 g) were used for the present study. Animals were assigned randomly to different groups. Thirty-six rats were decapitated at 2 h, 24 h, and 7 days after air or anesthesia, randomly divided into two groups for control or anesthesia at each time point (*n* = 6 in each group, [male = 20, female = 16]). And 48 rats were decapitated at 4 days after air or anesthesia, randomly divided into eight groups (*n* = 6 in each group, [male = 26, female = 22]). The DG regions of the hippocampus were harvested for western blot (*n* = 3 in each group) and brain sections were used for immunofluorescence (*n* = 3 in each group). Fifty rats were used for the Morris water maze (MWM) test at P28 (*n* = 10 in each group, [male = 28, female = 22]). Group numbers do not include 24 animals that died during anesthesia exposure, and 10 died during inhibitors injection.

### Sevoflurane Exposure

On P21, rats were randomized to fasting in 30% oxygen (O_2_)/70% nitrogen (N_2_) or anesthetized with 3% sevoflurane (about 1.5 MAC, Maruishi Pharmaceutical Co., Ltd., Japan, catalog: 95071) plus 30% O_2_/70% N_2_ for 4 h in control or anesthesia chambers. Inspired anesthetic and oxygen concentrations were monitored using a gas analyzer (RGM 5250; Datex-Ohmeda, Louisville, CO, United States). The temperature in the chambers was maintained via a heating pool at 37 ± 0.5°C. Rats rectal temperature were maintained at 37 ± 0.5°C. Rats for harvest at 2 h, 24 h, and 7 days time points post-treatment were assigned randomly into two study groups: (1) control groups and (2) anesthesia groups. Rats for harvest at the 4 days post-treatment time point were assigned randomly into eight study groups: (1) non-anesthesia + vehicle; (2) non-anesthesia + inhibitor of caspase-3; (3) non-anesthesia + inhibitor of LC3B; (4) non-anesthesia + inhibitor of NF-κB; (5) anesthesia + vehicle; (6) anesthesia + inhibitor of caspase-3; (7) anesthesia + inhibitor of LC3B; (8) anesthesia + inhibitor of NF-κB (*n* = 6 each group). Rats were sacrificed by intraperitoneal injection of 100 mg/kg pentobarbital (Sinopharm Chemical Reagent Co., Ltd., Shanghai, China, catalog: WS20051129).


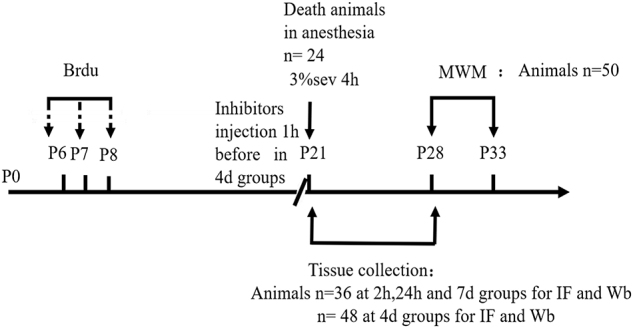


### Drug Administration

Rats in sevoflurane or control plus LC3B/caspase-3/NF-κB inhibitor groups were lateral ventricularly injected with 3-MA (50 μM Selleck Chemicals, Shanghai, China, Cat# S2767, purchased in 2017)/Q-VD-OPh (20 μM, Selleck Chemicals, Shanghai, China, Cat# S7311, purchased in 2017)/BAY11-7085 (40 μM, Selleck Chemicals, Shanghai, China, Cat# S7352, purchased in 2018). Drug administration was conducted 1 h before an exposure to anesthesia or 30% O_2_/70% N_2_ exposure. Vehicle only was also injected into the lateral ventricles of sevoflurane and control groups alone with the same volume of vehicle solution compared with inhibitor groups. For 5-bromo-2-deoxyuridine (BrdU) (Sigma–Aldrich, Cat# B50021G, catalog: HMBG3845V) administration, prior to anesthesia or 30% O_2_/70% N_2_ exposure, animals received BrdU (150 mg/kg) 13, 14, and 15 days before treatment (total of three doses).

### Tissue Preparation for Immunofluorescence

The rats were perfused through the ascending aorta with ice-cold PBS (0.1 M) for 30 s at 10 mL/min, immediately followed by a 4% paraformaldehyde in 0.1 M PBS at room temperature for 10 min. Brains were removed, and post-fixed overnight in phosphate-buffered PFA at 4°C. Brains for double immunofluorescence were cryoprotected in an ascending sucrose series (10, 20, and 30%) in PBS at 4°C, and were sectioned coronally at 10 μm on a cryostat. Brains for TUNEL and LC3B staining were sectioned coronally at 2.5 μm.

### TUNEL Immunofluorescence

The brain sections were incubated with terminal deoxynucleotidyl transferase (TdT) and dUTP (Roche, Basel, Switzerland, Cat# 11684795910, catalog: 30967400) simultaneously, at 37°C for 1 h. Nuclei were stained with 4’,6-diamidino-2-phenylindole (DAPI). Each brain section with the whole views of DG region, including the upper blade, lower blade, and crest of DG, was photographed with microscope (LSM 880) by an investigator who was blinded to the experimental interventions. The number of TUNEL/DAPI-positive cells was counted at 200× magnification by Image J software. The number for counting was from three continuous slides of each rat in the DG region of hippocampus. Area of DG was counted by Image J software. Density value of TUNEL in DG was quantified.

### Immunofluorescence Double Staining

Cryostat coronal sections (10 μm thick) were incubated with two kinds of primary antibodies (BrdU/NeuN or BrdU/DCX or Brdu/caspase3) simultaneously at 4°C overnight in an immunofluorescence double staining chamber. The slices were incubated with secondary antibodies the next day for 2 h at room temperature. The primary antibodies were Rabbit anti-NeuN (1:200, Abcam, Cat# ab177487, catalog: GR 249899-70), Rabbit anti-DCX (1:200, Cell Signaling Technology, Cat# 4604S, catalog: 5), Rabbit anti-caspase-3 (1:200, Cell Signaling Technology, Cat# NB100 56112, catalog: AR14-010410-07), and Mouse Anti-BrdU (1:200, novus, Cat# NBP2 32918, catalog: MSM1-469P170406). The secondary antibodies were goat anti-rabbit IgG (Alexa Fluor 488, 1:200, Abcam, Cat# ab150073, catalog: GR323256) and goat anti-mouse IgG (Alexa Fluor 594, 1:200, Abcam, Cat# ab150108, catalog: GR323518). The nuclei were stained with DAPI. The stained slices were observed with a Nikon C1 microscope by an investigator who was blinded to the group assignment of the sections. The whole fields of vision in the hippocampal DG region of the left cerebral hemispheres were selected per section to take photographs and cell counting. Quantification was performed using Image J software and positively double-labeled cells with BrdU/NeuN or BrdU/DCX or Brdu/caspase3, and BrdU-labeled cells in the DG were counted and expressed as a ratio of double-labeled cells with BrdU/NeuN or BrdU/DCX or Brdu/caspase3 to the total number of BrdU-labeled cells.

### Immunofluorescence of LC3B

The sections were incubated with Rabbit anti-LC3B (1:200, Abcam, Cat# ab48394, catalog: GR3241806-3) at 4°C overnight in a humidified chamber, and then incubated with secondary antibody, goat anti-rabbit IgG (Alexa Fluor 488, 1:200, Abcam, Cat# ab150073, catalog: GR32325). The nuclei were stained with DAPI. The stained slices were observed with a microscope by an investigator who was blinded to the group assignment of the sections. Each brain section with views of the upper blade, lower blade, and crest of DG region was photographed. Quantification was performed using Image ProPlus software to calculate the integrated optical density for LC3B in the DG region.

### Western Blot

The DG of the hippocampus was dissected at 2 h, 24 h, 4 days, and 7 days after anesthesia exposure. Tissues are collected and stored at −80°C and later used for western blot. DG regions for each rat were harvested to detect cleaved caspase-3, LC3B, P62/SQSTM1, NF-κB-P65, NF-κB−P−P65, and IκB protein expressions. The primary antibodies were Rabbit anti-cleaved caspase-3 (1:1000, Abcam, Cat# 13847, catalog: GR3230427-1), Rabbit anti−LC3B (1:1000, Abcam, Cat# ab48394, catalog: GR32418063), Rabbit anti−P62/SQSTM1 (1:1000, Abcam, Cat# ab109012, catalog: GR31991115), Rabbit anti−NF-κB-P65 (1:1000, Proteintech Biotechnology, Chicago, IL, United States, Cat# 10745-1-AP, catalog: 00064467), Rabbit anti−NF-κB−P−P65 (1:1000, Abcam, Cat# ab86299, purchased in 2018), and Rabbit anti-IκB (1:1000, Proteintech Biotechnology, Chicago, IL, United States, Cat# 10268-1-AP, catalog: 00080111). Rabbit GAPDH antibody (1:10,000, Proteintech Biotechnology, Chicago, IL, United States, Cat# 10494-1-AP, catalog: 00083125) was used as an internal loading control. The membranes were incubated with the following secondary antibody:goat anti-rabbit IgG secondary antibody (Zhongshanjinqiao Biotechnology Co., Ltd., Beijing, China) at room temperature for 2 h. The protein blots were detected and photographed by GE Amersham Imager 600 using ImageQuant 5.0 Windows NT software (Molecular Dynamics, Sunnyvale, CA, United States).

### Morris Water Maze

To test spatial learning and memory, 50 rats in five different groups (*n* = 10) were used in the MWM test. Probe trial sessions began at 8:00 am everyday. Rats were placed in the water, and subjected to four rounds of testing each lasting 90 s with a 30-min interval for 5 continuous days when they were P28 to P32 of age. Escape latency (the time taken to find the platform to escape the water) was recorded. If a rat found the platform within 90 s, it was made to sit on the platform for 20 s. If the rat was unable to find the platform within 90 s, it was guided to the platform for 20 s, and the escape latency was recorded as 90 s. For the probe trials on P33 after birth, the platform was removed, and platform crossings were recorded. An image acquisition and analysis system was used to record the frequency that the rat passed through the area of the original platform location within 90 s.

### Statistical Analysis

Data are presented as mean ± standard error. All continuous variables were tested for assumption of normality using the Shapiro–Wilk test. One-way analysis of variance (ANOVA) followed by the Student–Newman–Keuls *post hoc* test was used to compare the means of each group. Data that failed tests for either normality or equal variance were either transformed to normalize the data or compared using the Mann-Whitney rank sum test. The escape latency was tested by two-way repeated measures ANOVA followed by Dunnett’s multiple comparisons tests. For all analyses, statistical significance was determined using Graph Pad Prism 7.0 software (Graph Pad Software Inc., San Diego, CA, United States). Specific statistical tests used are noted in the results. Statistical significance was accepted for *P* < 0.05.

## Results

### Sevoflurane Exposure Affects the Differentiation of Late-Stage Progenitor Cells

**FIGURE 1:**
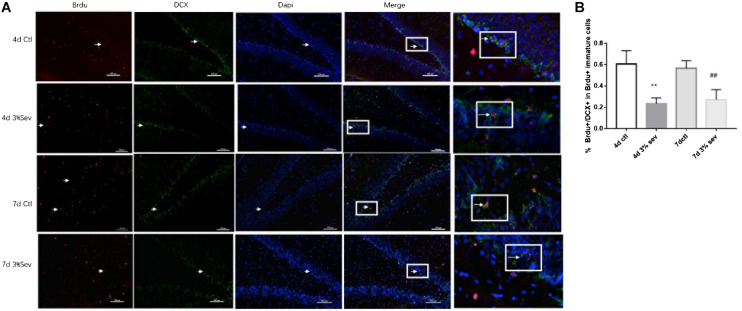
Sevoflurane exposure decreased the differentiation into DCX in BrdU+ immature cells 4 and 7 days after anesthesia. **(A)** BrdU (red) co-localization with DCX (green) presented the late progenitor cells differentiating into immature cells in the 4 and 7 days in sevoflurane and control groups. Scale bar = 100 μm. **(B)** Quantification percentage of BrdU+/DCX+ in BrdU+ immature cells. Values are presented as mean ± SD, *n* = 3; **P < 0.01 compared with the control 4 days group, ##*P* < 0.01 compared with the control 7 days group. One-way ANOVA with Newman–Keuls *post hoc* test or Kruskal–Wallis with Dunn’s Multiple comparison test was used for data analysis.

Adult hippocampal neurogenesis originates from a population of neuronal precursor cells in the SGZ (Deng et al., 2010), which give rise to intermediate progenitor cells with neuronal phenotype which take up residence in the inner third of the granule cell layer (Mathews et al., 2010; Kempermann et al., 2015). The developmental stages of newborn DGCs can be distinguished on the basis of the expression of stage-specific marker proteins, including early progenitor cells (Sox2), immature cells (DCX), and mature cells (NeuN). For identification of late-stage progenitor cells during anesthetic exposure, animals were injected with the S-phase marker BrdU to birth-date late-stage progenitor cells 13, 14, and 15 days prior to anesthetic exposure on P21 (Hofacer et al., 2013). As such, double-stained BrdU and DCX cells represent the cohort of immature granule cells differentiated from 14-day-old cells. The ratio of double-immunopositive (BrdU+/DCX+) cells of all the BrdU positive cells of 14-day age in the SGZ and the inner third of the granule cell body layer were measured. We observed that the ratio decreased in the first week after treatment, on P25 and P28 (Figures 1, 4 days *P* = 0.0020 and 7 days *P* = 0.0077). These results indicated that, compared to control, sevoflurane on P21 decreased the number of immature neurons in DG differentiated from 14-day-old cells.

### Effects of Sevoflurane on Short-Term Differentiation From a Cohort of Late-Stage Progenitor Cells

**FIGURE 2:**
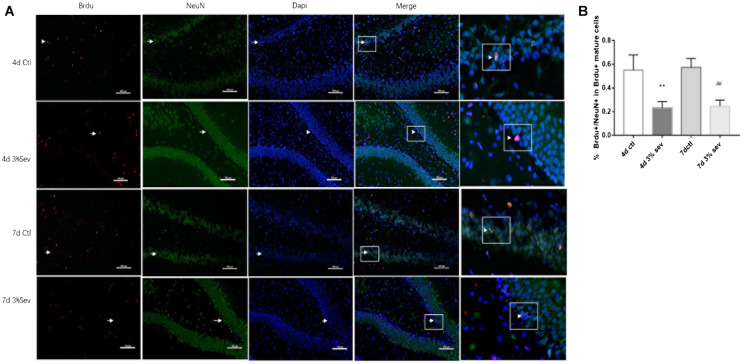
Sevoflurane exposure deregulated differentiation into neuron in BrdU+ mature cells 4 and 7 days after anesthesia. **(A)** BrdU (red) co-localization with NeuN (green) presented the late progenitor cells differentiating into mature cells in the 4 and 7 days in sevoflurane and control groups. Scale bar = 100 μm. **(B)** Quantification percentage of BrdU+/NeuN+ in BrdU+ mature cells. Values are presented as mean ± SD, *n* = 3; **P < 0.01 compared with the control 4 days group, ##*P* < 0.01 compared with the control 7 days group. One-way ANOVA with Newman–Keuls *post hoc* test or Kruskal–Wallis with Dunn’s Multiple comparison test was used for data analysis.

Newborn mature DGCs can be identified by the presence of a mature neuronal marker (NeuN) (Kuhn et al., 2016). In our study, we used double-staining for BrdU+/NeuN+ to represent cells that have differentiated into mature newborn granule cells. The ratio of double-immunopositive (BrdU+/NeuN+) cells of all BrdU positive cells in the granule layer (GL) was measured. We found that sevoflurane reduced the differentiation ratio 4 and 7 day after exposure (Figures 2, 4 days *P* = 0.0033 and 7 days *P* = 0.0029). These results indicated that sevoflurane decreased the process of differentiation from late-stage progenitor cells to newly mature generated neurons in the DG.

In summary, the results suggest that sevoflurane decreases differentiation of late progenitor granule cells into both immature and mature cells in DG region.

### Sevoflurane Increases Short-Term Apoptotic Cell Death and Represses Differentiation of Late-Stage Progenitor Cells

**FIGURE 3:**
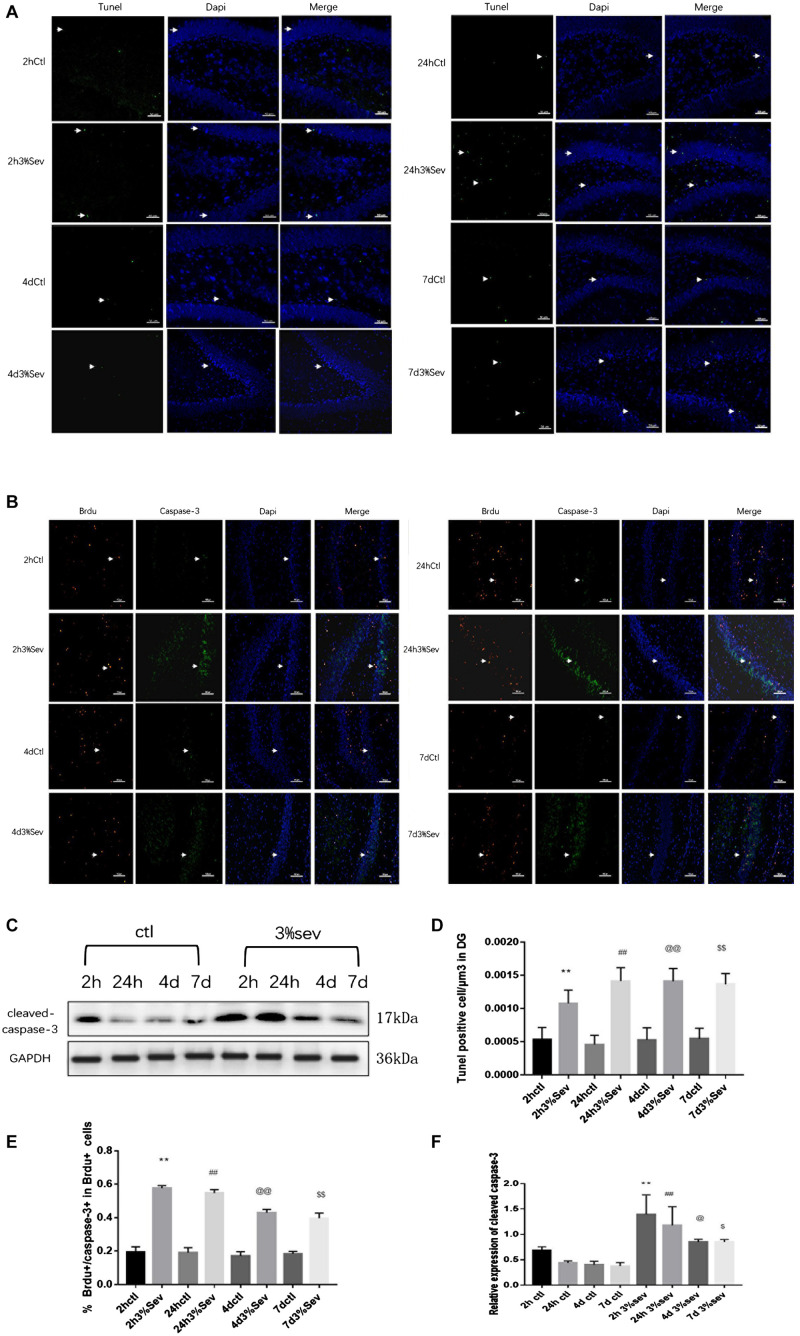
**(A)** Sevoflurane increased TUNEL expression in DG region 2 h, 24 h, 4 days, and 7 days after anesthesia. Scale bar = 50 μm. **(B)** BrdU (red) co-localization with caspase-3 (green) presented the apoptotic late progenitor cells 2 h, 24 h, 4 days, and 7 days in sevoflurane and control groups. Scale bar = 100 μm. **(C)** Sevoflurane increased cleaved caspase-3 expression in DG region 2 h, 24 h, 4 days, and 7 days after anesthesia. **(D)** Quantification of TUNEL positive cells/μm^3^ in DG region. **(E)** Quantification percentage of BrdU+/caspase-3+ in BrdU+ cells. **(F)** Quantification of cleaved caspase-3. *n* = 3. Values are presented as mean ± SD; **P < 0.01 compared with the 2-h control group, ##*P* < 0.01 compared with the 24-h control group, @*P* < 0.05 compared with the 4 days control group, @@*P* < 0.01 compared with the 4 days control group, $*P* < 0.05 compared with the 7 days control group, $$*P* < 0.01 compared with the 7 days control group.

**FIGURE 4:**
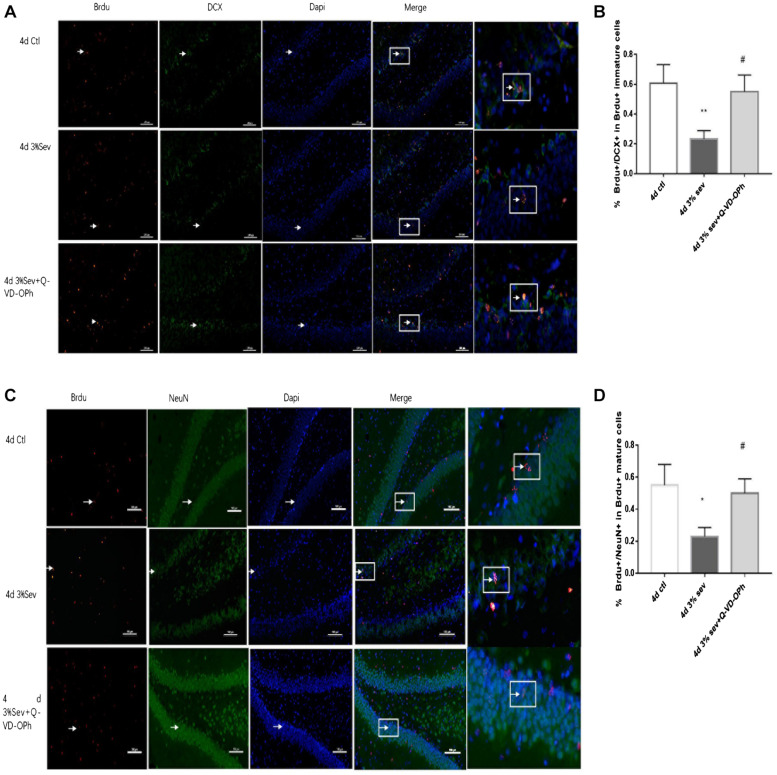
**(A)** Caspase-3 inhibitor alleviated sevoflurane-induced decrease of differentiation into DCX in BrdU+ immature cells 4 days after anesthesia. **(C)** Caspase-3 inhibitor alleviated sevoflurane-induced decrease of differentiation into NeuN in BrdU+ mature cells 4 days after anesthesia. Scale bar = 100 μm. **(B)** Quantification percentage of BrdU+/DCX+ in BrdU+ immature cells. **(D)** Quantification percentage of BrdU+/NeuN+ in BrdU+ mature cells. Values are presented as mean ± SD, *n* = 3; *P < 0.05 compared with the control 4 days group, **P < 0.01 compared with the control 4 days group, #*P* < 0.05 compared with the 4 days sevoflurane group. One-way ANOVA with Newman–Keuls *post hoc* test or Kruskal–Wallis with Dunn’s Multiple comparison test was used for data analysis.

Previous studies demonstrated vulnerability to anesthesia-induced neuroapoptosis in DG cells on P21 (Deng et al., 2014). In order to test the short-time change on neuronal apoptosis in DG cells, we tested cleaved caspase-3 levels in DG region, and found an increased cleaved caspase-3 (Figures 3C,F, 2 h *P* = 0.0016, 24 h *P* = 0.0011, 4 days *P* = 0.0481, and 7 days *P* = 0.0357). We also observed a consistent increase in the number of TUNEL-positive cells in DG (Figures 3A,D 2 h *P* = 0.0085, 24 h *P* < 0.001, 4 days *P* < 0.001, and 7 days *P* = 0.0002). In addition, double-staining for BrdU+/caspase3+ represents the apoptosis of newly-born 14-day cells in DG induced by sevoflurane treatment, and the ratio of double-immunopositive (BrdU+/caspase3+) cells of all BrdU positive cells in the GL was measured. We found that sevoflurane induced apoptosis in newly-born granule cells of 14 days old after exposure (Figures 3B,E, 2 h *P* < 0.001, 24 h *P* < 0.001, 4 days *P* < 0.001, and 7 days *P* < 0.001). In order to prove that differentiation was mediated by apoptosis, the caspase-3 inhibitor was injected into P21 rats 1 h before gas treatment. We found an increase in the ratio of DCX and NeuN differentiated from BrdU labeled 14-day-old granule cells compared with sevoflurane alone (Figures 4A,B *P* = 0.0207 and Figures 4C,D, *P* = 0.0328).

### Sevoflurane Exposure Increases Autophagy and Represses the Differentiation of Late-Stage Progenitor Cells

**FIGURE 5:**
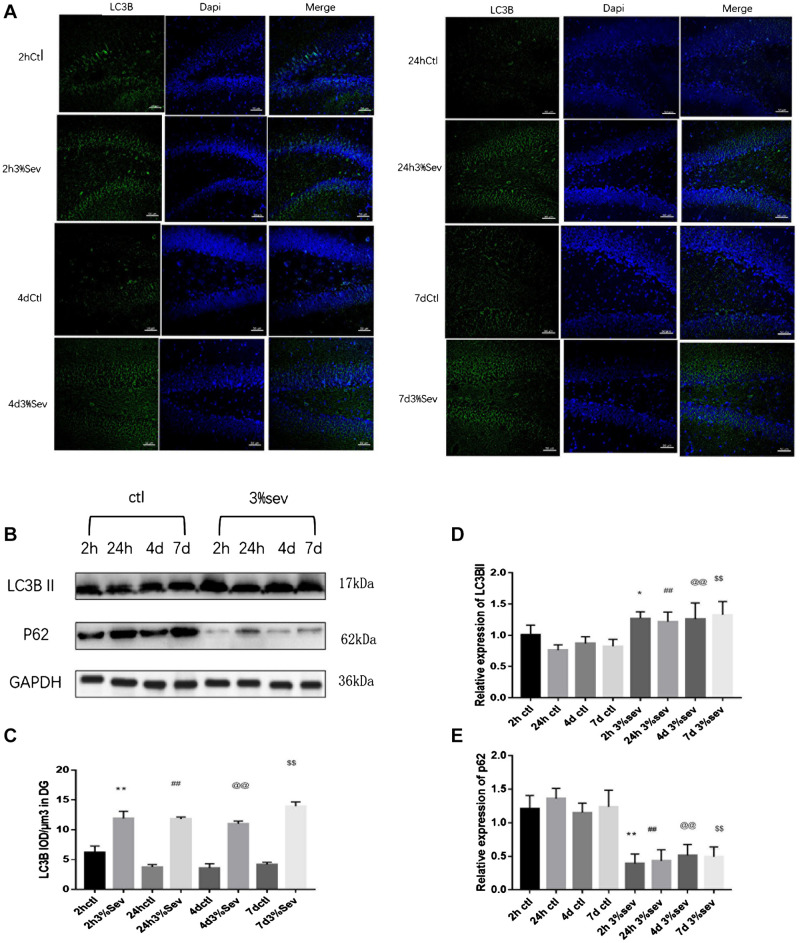
**(A)** Sevoflurane increased LC3B expression in DG region 2 h, 24 h, 4 days, and 7 days after anesthesia. Scale bar = 50 μm. **(B)** Sevoflurane upregulated LC3BII and decreased P62 expression in DG region 2 h, 24 h, 4 days, and 7 days after anesthesia. **(C)** Quantification analysis of the fluorescence intensity of LC3B/μm^3^ in the DG. *n* = 3. **(D)** Quantification of LC3BII. **(E)** Quantification of P62. *n* = 3. Values are presented as mean ± SD; *P < 0.05 compared with the 2-h control group, **P < 0.01 compared with the 2-h control group, ##*P* < 0.01 compared with the 24-h control group, @@*P* < 0.01 compared with the 4 days control group, $$*P* < 0.01 compared with the 7 days control group. One-way ANOVA with Newman–Keuls *post hoc* test or Kruskal–Wallis with Dunn’s Multiple comparison test was used for data analysis.

**FIGURE 6:**
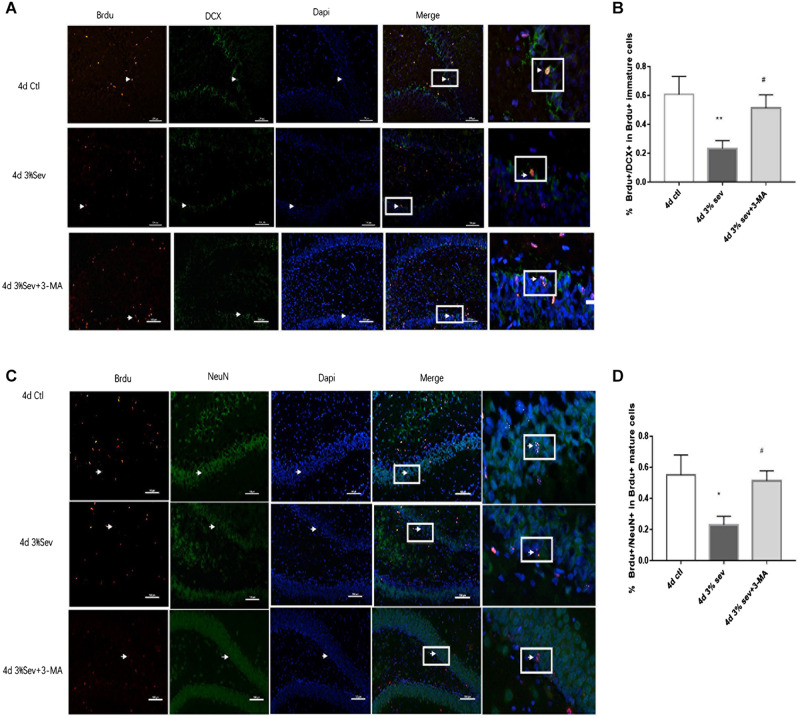
**(A)** LC3B inhibitor alleviated sevoflurane-induced decrease of differentiation into DCX in BrdU+ immature cells 4 days after anesthesia. **(C)** LC3B inhibitor alleviated sevoflurane-induced decrease of differentiation into NeuN in BrdU+ mature cells 4 days after anesthesia. **(B)** Quantification percentage of BrdU+/DCX+ in BrdU+ immature cells. **(D)** Quantification percentage of BrdU+/NeuN+ in BrdU+ mature cells. Values are presented as mean ± SD, *n* = 3; *P < 0.05 compared with the control 4 days group,**P < 0.01 compared with the control 4 days group, #*P* < 0.05 compared with the 4 days sevoflurane group. One-way ANOVA with Newman–Keuls *post hoc* test or Kruskal–Wallis with Dunn’s multiple comparison test was used for data analysis.

Autophagy, as another manner of cell death, plays a functional role in neural differentiation. In our study, western blot results suggested that autophagy levels changed after sevoflurane exposure. In the current study, we found a large increase expression in autophagy in DG at 2 h post-treatment (in Figures 5B,D, *P* = 0.0197). This effect was sustained after 24 h, 4 days, and 7 days (in Figures 5B,D, 24 h *P* = 0.0036, 4 days *P* = 0.001, and 7 days *P* = 0.0014). We also tested the expression of P62/SQSTM1, which is specially degraded in autolysosomes, the results showed that the expression of P62/SQSTM1 decreased at 2 h, 24 h, 4 days, and 7 days (in Figures 5B,E, 2 h *P* = 0.001, 24 h *P* < 0.001, 4 days *P* = 0.0015, and 7 days *P* = 0.0003). In addition, we measured the fluorescence intensity of LC3B in DG, and the results of 2 h, 24 h, 4 days and 7 days consisted with those seen by western blot, with an obvious increase in autophagy at 2 h, 24 h, 4 days, and 7 days (in Figures 5A,C 2 h *P* < 0.001, 24 h *P* < 0.001, 4 days *P* < 0.001, and 7 days *P* < 0.001). To observe the role of autophagy in neural differentiation, 3-MA was injected 1 h before anesthesia. Tissues were collected for morphological study 4 days after anesthesia. Autophagy inhibition with 3-MA increased in the rate of DCX and NeuN fluorescence (in Figures 6A,B, *P* = 0.0238 and Figures 6C,D *P* = 0.0196). In summary, it is clear that anesthesia-activated autophagy could decrease the differentiation of 14-day-old granule cells during adult neurogenesis.

### Apoptosis Induced by Sevoflurane Is Mediated by Autophagy

**FIGURE 7:**
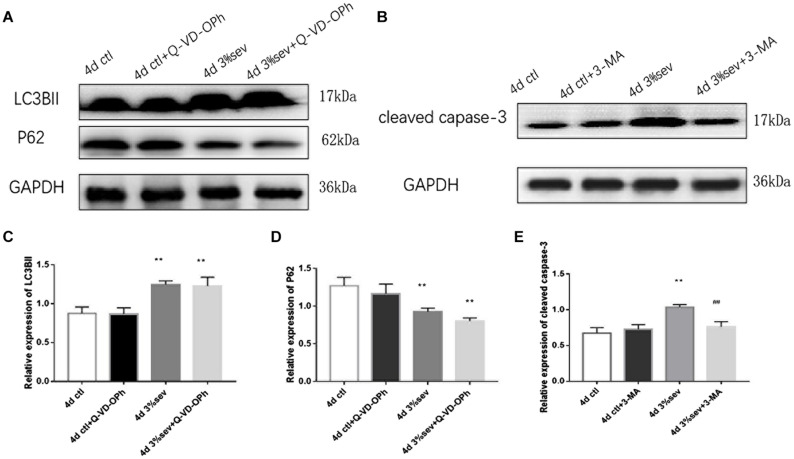
**(A)** LC3B inhibitor alleviated sevoflurane-induced cleaved caspase-3 expression in DG region 4 days after anesthesia. **(B)** Caspase-3 inhibitor did not affect sevoflurane-induced LC3BII expression in DG region 4 days after anesthesia. **(C)** Quantification of LC3BII. **(D)** Quantification of P62. **(E)** Quantification of cleaved caspase-3. *n* = 3. Values are presented as mean ± SD; **P < 0.01 compared with the control 4 days group, ##*P* < 0.01 compared with the 4 days sevoflurane group. One-way ANOVA with Newman–Keuls *post hoc* test or Kruskal–Wallis with Dunn’s multiple comparison test was used for data analysis.

The results above (Figures 3–5) showed increases in both autophagy and apoptosis were observed at 2 h, 24 h, 4 days, and 7 days after exposure. Thus, we conducted further observations to investigate if there was relationship between autophagy and apoptosis. Autophagy and apoptosis inhibitors were used for comparisons in sevoflurane alone group. In the sevoflurane + autophagy inhibitor group, cleaved caspase-3 level was reduced compared with sevoflurane alone (Figures 7B,E, *P* = 0.0034); however, in the sevoflurane + apoptosis inhibitor group, LC3BII and P62 were not affected compared with sevoflurane alone (Figures 7A,C,D *P* = 0.9933, *P* = 0.4091). This results implied that the apoptosis induced by sevoflurane was mediated by autophagy activation. However, autophagy was not be mediated by apoptosis induced by 3% sevoflurane.

### Sevoflurane Enhances Autophagy and Apoptosis by NF-κB Signaling

**FIGURE 8:**
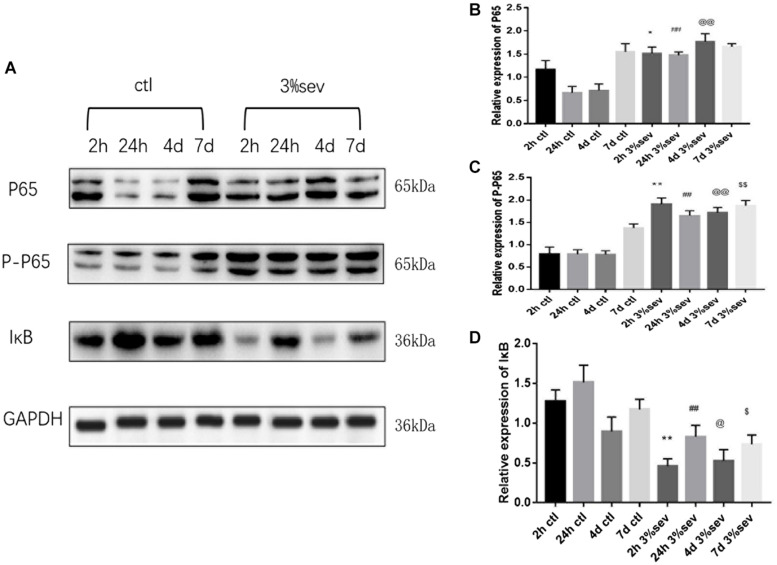
**(A)** Sevoflurane upregulated both P65 and P-P65 expression in DG region 2 h, 24 h, and 4 days after anesthesia, but only P-P65 expression 7 days after anesthesia. In contrast, sevoflurane decreased IκB expression in DG 2 h, 24 h, 4 days, and 7 days after anesthesia. **(B)** Quantification of P65. **(C)** Quantification of P-P65. **(D)** Quantification of IκB. *n* = 3. Values are presented as mean ± SD, *n* = 3; *P < 0.05 compared with 2 h control group, **P < 0.01 compared with 2 h control group, ##*P* < 0.01 compared with 24 h control group, @*P* < 0.05 compared with 4 days control group, @@*P* < 0.01 compared with 4 days control group, $*P* < 0.05 compared with 7 days control group, $$*P* < 0.01 compared with 7 days control group. One-way ANOVA with Newman–Keuls *post hoc* test or Kruskal–Wallis with Dunn’s Multiple comparison test was used for data analysis.

**FIGURE 9:**
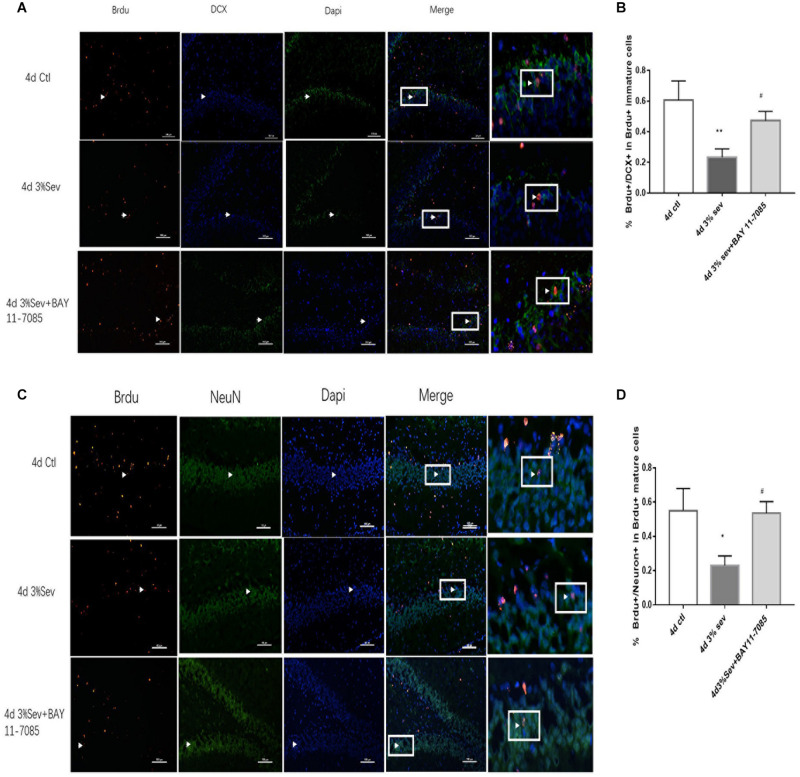
**(A)** NF-κB inhibitor alleviated sevoflurane-induced decrease of differentiation into DCX in BrdU+ immature cells 4 days after anesthesia. **(B)** NF-κB inhibitor alleviated sevoflurane-induced decrease of differentiation into NeuN in BrdU+ mature cells 4 days after anesthesia. Scale bar = 100 μm. **(C)** Quantification (percentage) of BrdU+/DCX+ in BrdU+ immature cells. **(D)** Quantification (percentage) of BrdU+/NeuN+ in BrdU+ mature cells. *n* = 3. Values are presented as mean ± SD; *P < 0.05 compared with 4 days control group, **P < 0.01 compared with the 4 days control group, #*P* < 0.05 compared with 4 days sevoflurane group.

**FIGURE 10:**
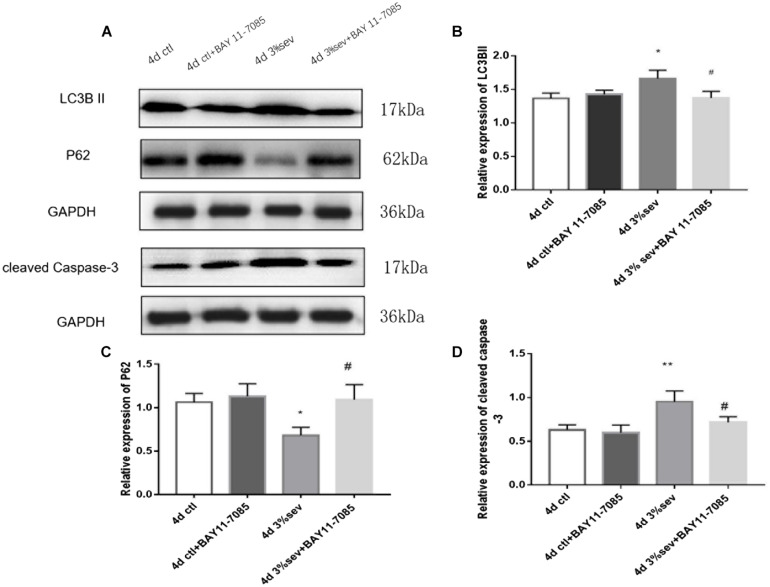
**(A)** NF-κB inhibitor alleviated sevoflurane-induced LC3BII and cleaved caspase-3, and increased P62 expressions in DG region 4 days after anesthesia. **(B)** Quantification of LC3BII. **(C)** Quantification of P62. **(D)** Quantification of cleaved caspase-3. *n* = 3. Values are presented as mean ± SD; *P < 0.05 compared with 4 days control group, **P < 0.01 compared with 4 days control group, #*P* < 0.05 compared with 4 days sevoflurane group.

First, we investigated IκB/NF-κB/P65 activity using western blot at 2 h, 24 h, 4 days, and 7 days after sevoflurane exposure. Levels of P65, P-P65 (phosphorylated-P65), and IκB were measured after sevoflurane exposure (Figures 8A–D). The results showed that sevoflurane significantly upregulated P65 at 2 h, 24 h, and 4 days timepoints and P-P65 levels at all post exposure time points (P65 in Figures 8A,B, 2 h *P* = 0.038, 24 h *P* < 0.001, 4 days *P* < 0.001, and 7 days *P* = 0.835), (P-P65 in Figures 8A,C, 2 h *P* < 0.001, 24 h *P* < 0.001, 4 days *P* < 0.001, and 7 days *P* = 0.0003). For the expression of IκB, the result of all post exposure timepoints showed a decrease in sevoflurane compared with control groups (Figures 8A,D, 2 h *P* < 0.001, 24 h *P* = 0.0002, 4 days *P* = 0.0329, and 7 days *P* = 0.0105). In sevoflurane + BAY11-7085 group, ratios of both DCX and NeuN in 14-day-old cells were increased (Figures 9A,B *P* = 0.0331 and Figures 9C,D *P* = 0.014). To investigate the mechanism of autophagy induced by IκB/NF-κB signaling after sevoflurane exposure, we examined the level of LC3B in the sevoflurane + BAY11-7085 group. Compared with sevoflurane group, LC3BII and cleaved caspase-3 levels were significantly declined in sevoflurane + BAY11-7085 group (Figures 10A,B *P* = 0.0247 and Figures 10A,D *P* = 0.0423), and P62 level was significantly increased in NF-κB inhibitor group (Figures 10A,C *P* = 0.022).

### Sevoflurane Exposure Leads to Short-Term Cognitive Deficiency

**FIGURE 11:**
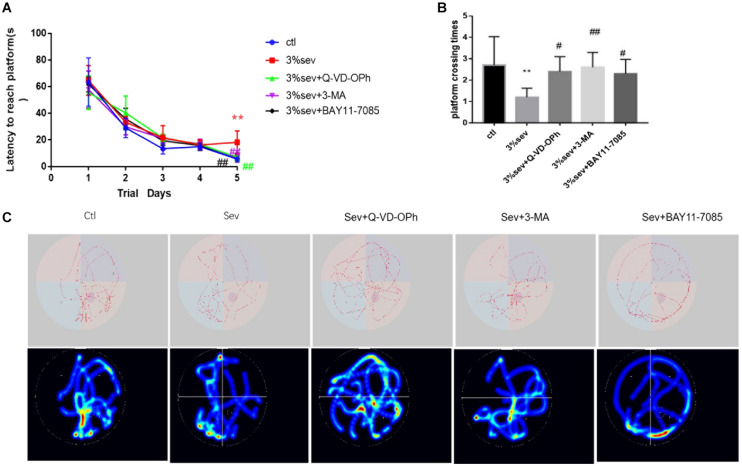
Sevoflurane reduced short-term cognitive deficiency via NF-κB/P65/autophagy/apoptosis regulation. **(A)** Escape latency in place trials evaluated the acquisition of spatial information. **(B)** Platform crossing times in probe test evaluated the memory retention ability. **(C)** Representative traces and graphical search patterns during MWM tests. Values are presented as mean ± SD; two-way repeated ANOVA followed by Dunnett’s multiple comparisons tests was used for escape latency. **P < 0.01 compared with the control group, #*P* < 0.05 compared with sevoflurane group, ##*P* < 0.01 compared with sevoflurane group.

The MWM test was performed between control and sevoflurane groups from P28-P33 to investigate the effects of sevoflurane exposure on spatial learning and memory (Figure 11). On P32, rats in the control group spent less time in escape latency compared with the sevoflurane group (Figure 11A, *P* < 0.001), which suggests that 3% sevoflurane may cause cognitive deficits. Further, to verify NF-κB, autophagy, and apoptosis mediated short-term neurological deficits in rats after sevoflurane exposure, the escape latency among the groups was measured. The results showed that the three inhibitors (NF-κB, autophagy, apoptosis) investigated all significantly shortened the escape latency compared to the sevoflurane alone group on P32 (Figure 11A, *P* = 0.002, *P* = 0.001, *P* = 0.001). This suggests that the three inhibitors investigated could improve the neurological deficits caused by sevoflurane. Rats in sevoflurane alone group crossed the platform fewer times than the control group on P33 (Figure 11B, *P* = 0.0017), which also suggests that exposure to sevoflurane disorientated the rats and affected their spatial memory. Results from sevoflurane + the three inhibitors groups (apoptosis, autophagy, NF-κB) indicated that apoptosis, autophagy, and NF-κB inhibitors could improve the spatial memory deficits caused by sevoflurane (Figure 11B, *P* = 0.0175, *P* = 0.0038, *P* = 0.0354). The results (in Figure 11C) showed the representative traces and graphical search patterns during MWM tests among the groups.

## Discussion

In the adult mammalian brain, neurogenesis continues throughout life in the DG of the hippocampus. Only one type of neurons, the DGC is generated (Kempermann et al., 2015). The neurogenesis of new born DG cells can be divided into four phases: precursor cell phase, early survival phase, postmitotic maturation phase, and late survival phase (Kempermann et al., 2015). In the early survival phase, the late-stage progenitor granule cells appear to promote neuronal differentiation and play a key role in generating newborn immature as well as maturing into adult granule cells. Given the importance of late-stage progenitor hippocampal granule cells in the process of neurogenesis, we tested the effects of the sevoflurane, a commonly used GA in pediatric surgery on their differentiation into immature neurons and their maturation into adult DG neurons. We further evaluated the behavior outcome of sevoflurane exposure on developing rats. Our results indicated that sevoflurane decreased rate of differentiation of late stage progenitor cells, likely mediated by apoptosis. Moreover, autophagy was found to regulate apoptosis.

Previous studies showed that anesthetic treatment could inhibit the process of neurogenesis in the DG. It has been shown that during early postnatal period, the stem cell pool was inhibited by sevoflurane and that this inhibitory effect could last until adulthood (Park et al., 2017; Shao and Xia, 2019). Treatment with propofol, an intravenous anesthetic with a similar mechanism of action as sevoflurane, on P7–P9 was shown to reduce the numbers of newly formed neurons in DG on P17. The reduction in cell number was accompanied by a delay in dendritic spine maturation of DG neurons (Huang et al., 2016). Our findings are consistent with their findings in that the process of adult neurogenesis at the late survival phase was found to be delayed. Since the differentiation into immature and the maturation of these neurons take 1–2 weeks in rodents models, our study established a time window of 4 and 7 days after anesthesia (P25 and P28) exposure in order for the delay to be observed. Our results also provide an answer whether longer anesthetic (4 h) decreased neurogenesis in the short term after anesthesia. Our results would suggest that P21 in rodents is not equivalent to adulthood in humans, since in our previous study (where anesthetic was administered on P21), there was no measurable effects of isoflurane on adult neurogenesis. However, it indicates that on P21 in rodents, neurogenesis is still ongoing, with new granule cells replacing the degenerated cells and possibly forming new connections to different regions of the brain.

In DG, most apoptotic cells are detected in the SGZ, the border between the granule cell layer and hilus, where the dividing progenitors reside (Seki, 2002). In our prior work, we demonstrated that the vulnerable period of apoptosis caused by anesthetic occurred 1 week after the peak time of adult neurogenesis. A high number of apoptotic granule cells were found in the DG immediately after anesthesia in the 21-day-old mouse model. Late-stage progenitor granule cells usually are defined as the newly generated cells within 14 days (Hofacer et al., 2013). Here, we found that apoptosis of newly-born late progenitor cells was increased for 1 week after a 4 h exposure to sevoflurane exposure. Although the exposure time in our study was shorter than in the prior study (4 vs 6 h), a 4-h exposure duration was selected based on clinical data, which showed that significant learning deficits in children are associated with >2 h of anesthetic exposure (Wilder et al., 2009). In addition, studies suggest that 4 h of exposure to 3% sevoflurane (1.5 MAC) induces excessive apoptotic injury in rodents compared with untreated animals (Fang et al., 2012; Zheng et al., 2013). Apoptotic cells dynamically regulated the survival and differentiation of newly-born neurons in the adult hippocampus. Thus, further work was undertaken to explore the influence of sevoflurane-induced apoptosis on the process of differentiation into immature and mature cells from late progenitor granule cells. Our results confirmed the findings that *in vivo* treatment with caspase-3 inhibitor increased the survival rate of newly generated neurons (Biebl et al., 2005). As such, we conclude that sevoflurane-induced apoptosis in DG influences differentiation of newly-born neurons in the hippocampus.

To explore the potential mechanisms of apoptosis after sevoflurane exposure, and its effects on differentiation from late-stage progenitor cells in DG region, we examined autophagy (both immunofluorescence and western blot) during the same time window of apoptosis. It is reported that autophagy plays a crucial role in the process of the neural maturation. Under normal condition, autophagy is thought to control stem cell fate by regulating the proliferative type of neural precursor cells, which has a homeostatic role as an energy provider during the early stages of neuronal differentiation (Benito-Cuesta et al., 2017). Li et al. (2017) reported that 3.5% sevoflurane could induce autophagy of neural stem cells. Much remains to be understood about the specific functions of autophagy during the multiple stages of adult neurogenesis, such as the late survival phase and its role in differentiation of late-stage progenitor granule cells. In our study, after longer exposed to 3% sevoflurane in P21 rats, we found autophagy increased starting from 2 h after exposure, and continue until P28. During the same period, apoptosis is also evident, coincidentally, differentiation into mature cells was also delayed. In this study, we were interested in evaluating whether autophagy is affected by sevoflurane altered differentiation of late-stage progenitor granule cells in the SGZ. When 3-MA was used to block the autophagic flux, the results showed that the rate of newly-born mature granule cells generated from 14-day-old late-stage progenitor granule cells was increased. Our results indicated that the increased in autophagy by anesthetic may contribute play a role in regulating adult neurogenesis.

Apoptosis and autophagy in DG appeared to reduce the survival of mature neurons; however, it is unknown which process played the leading role. Studies have found crosstalk between the two processes, whereby autophagy controls apoptosis, and apoptosis-associated caspase activation shuts off the autophagic process (Li et al., 2019). It is widely accepted that the interaction between apoptosis and autophagy is complex. In physiological state, autophagy generally blocks the induction of apoptosis. However, in special circumstances, such as during general anesthesia treatment, autophagy or autophagy-relevant proteins may help to induce apoptosis. One study reported that autophagy of neural stem cells activated by 3% sevoflurane could induce apoptosis in the hippocampus (Li et al., 2017). However, the interactions between autophagy and apoptosis remain undetermined. Here, we conducted tests using inhibitors of autophagy and caspase-3 prior to sevoflurane treatment and found that apoptosis was reduced with 3-MA pretreatment, whereas autophagy levels remained high despite caspase-3 inhibitor pretreatment. These results imply that the increased apoptosis in DG after 3% sevoflurane exposure was influenced by activated autophagy, while activated autophagy was not induced by apoptosis. Our findings contradict the finding of other laboratory which reported that caspase cleavage of apoptosis-related proteins can affect the autophagic process (Li et al., 2019). The reason for the conflicting results might be that the pathological conditions were different (sevo exposure vs Cd-induced). Taken together, our results indicate that both activated autophagy and apoptosis could control the process of late-stage progenitor granule cells differentiation into immature and mature granule cells, and that the action of autophagy was indirect following the upregulation of apoptosis.

The signaling pathway mediating the crosstalk between activated autophagy and apoptosis remains unclear. It has been reported that NF-κB interacts with autophagy, leading to alterations in cell survival and apoptosis (Dresselhaus and Meffert, 2019), as well as regulating proliferation and apoptosis of neural progenitor cells and nascent neurons maturation (Widera et al., 2008). NF-κB inhibition also promotes survival and neuronal differentiation of transplanted NSCs (Rolls et al., 2007). We decided to examine the role of NF-κB signaling in sevoflurane induced neurotoxicity. First, we measured the protein levels of P65 and its phosphorylated derivative P-P65. They belong to the NF-κB family subunits and are synthesized as mature proteins. Using western blot, we found that the attenuation of NF-κB nuclear phosphorylation of P-P65 follows a similar time course of changes in autophagy and apoptosis after exposure to 3% sevoflurane. Our results showed that the level of IκB continuously decreased, indicating that sevoflurane could suppress IκB, which can lead to activation of NF-κB/P65 phosphorylation. To investigate the role of NF-κB signaling in neuronal differentiation during adult neurogenesis after sevoflurane exposure, we determine if blockade of NF-κB activation would rescue the disruptions of late-stage progenitor granule cells differentiation. We used the NF-κB inhibitor, BAY11-7085 which is known to inhibit the phosphorylation of IκB (Shao et al., 2016). We found that the ratio of DCX to NeuN in BrdU labeled 14-day-old granule cells (Figure 9) was increased by BAY 11-7085. These results indicated that delayed maturation of DG cells by sevoflurane can be affected by NF-κB levels, supporting the findings that NF-κB inhibitor could promote the survival of neural stem cells and differentiation of neurons. We also investigated whether NF-κB signaling was involved in the regulation of autophagy and apoptosis by sevoflurane. Our results (Figure 10) showed that autophagy and apoptosis decreased in NF-κB inhibitor treated group, indicating that apoptosis activation by sevoflurane-induced autophagy via the IκB/NF-κB pathway. This is in line with the finding that activation NF-κB could indeed promote apoptosis (Abhari et al., 2019), though an anti-apoptotic role of NF-κB has also been reported (Shen et al., 2013).

We evaluated the short-term cognitive effects of 3% sevoflurane exposure for 4 h on P21 rats with the use of the MWM test. Studies were performed on animals 7–14 days after sevoflurane exposure. The results (Figure 11) indicated that sevoflurane caused cognitive dysfunction by prolonging escape latency on P32 and platform crossing times on P33. The results could answer the question that whether cognitive and behavioral functions were effected after sevoflurane exposure in P21 rodents models. Cognitive, learning, and memory ability postnatally has a close relationship with adult neurogenesis, especially with regard to newly generated mature DGCs (Lacefield et al., 2012). Our results have showed that autophagy, apoptosis, and NF-κB were involved in the differentiation of late-stage progenitor cells. To better understand whether increased autophagy, apoptosis, and NF-κB contribute to sevoflurane-induced cognitive impairments in P21 rats, inhibitions of autophagy, apoptosis, and NF-κB were used in the MWM test. Previous studies suggested that NF-κB/autophagy/apoptosis inactivation could suppress sevoflurane-induced adult neurodegeneration and alleviate the cognitive dysfunction (Xiao et al., 2017; Zheng et al., 2017). Here, we also found that inhibitions of autophagy, apoptosis, and NF-κB all improved short-term cognitive dysfunction induced by sevoflurane. Based on our findings, we propose that inactivation of NF-κB/autophagy/apoptosis play neuroprotective effects to resist sevoflurane-induced neurotoxicity.

## Conclusion

Our results demonstrated that a single, prolonged exposure to sevoflurane could impair the differentiation of this age-specific cohort of DGCs in the hippocampus. This may lead to a deficit in cognitive ability possibly via apoptosis activated by autophagy through NF-κB signaling. We suggest that suppressions of apoptosis, autophagy, and NF-κB signaling in DG could be potential therapeutic targets for the treatment of sevoflurane-induced neurotoxicity.

## Data Availability Statement

The original contributions presented in the study are included in the article/supplementary material. Further inquiries can be directed to the corresponding author/s.

## Ethics Statement

The animal study was reviewed and approved by the Institutional Animal Care and Use Committee of the Shengjing Hospital Research Foundation (Approval No. 2016PS138K).

## Author Contributions

DT and PZ conceived and designed the experiments. DT, ZM, PS, LZ, and YX performed the experiments and generated and analyzed the data. DT wrote the manuscript with the help of SW, ZW, and KL. All authors read and approved the final manuscript.

## Conflict of Interest

The authors declare that the research was conducted in the absence of any commercial or financial relationships that could be construed as a potential conflict of interest.
